# Maturation of monocyte derived dendritic cells with OK432 boosts IL-12p70 secretion and conveys strong T-cell responses

**DOI:** 10.1186/1471-2172-12-2

**Published:** 2011-01-05

**Authors:** Arnt-Ove Hovden, Marie Karlsen, Roland Jonsson, Hans Jørgen Aarstad, Silke Appel

**Affiliations:** 1 Broegelmann Research Laboratory, The Gade Institute, University of Bergen, Laboratory Building, 5th floor west, N-5021 Bergen, Norway; 2 Department of Surgical Sciences, University of Bergen, Norway; 3 Department of Head and Neck Surgery, Haukeland University Hospital, Bergen, Norway

## Abstract

**Background:**

Design of tumour specific immunotherapies using the patients' own dendritic cells (DC) is a fast advancing scientific field. The functional qualities of the DC generated *in vitro *are critical, and today's gold standard for maturation is a cytokine cocktail consisting of IL-1β, IL-6, TNF-α and PGE_2 _generating cells lacking IL-12p70 production. OK432 is an immunotherapeutic agent derived from killed *Streptococcus pyogenes *that has been used clinically to treat malignant and benign neoplasms for decades.

**Methods:**

In this study, we analysed the effects of OK432 on DC maturation, DC migration, cytokine and chemokine secretion as well as T-cell stimulatory capacity, and compared it to the cytokine cocktail alone and combinations of OK432 with the cytokine cocktail.

**Results:**

OK432 induced a marked up-regulation of CD40 on the cell surface as well as a strong inflammatory response from the DC with significantly more secretion of 19 different cytokines and chemokines compared to the cytokine cocktail. Interestingly, secretion of IL-15 and IL-12p70 was detected at high concentrations after maturation of DC with OK432. However, the OK432 treated DC did not migrate as well as DC treated with cytokine cocktail in a transwell migration assay. During allogeneic T-cell stimulation OK432 treated DC induced proliferation of over 50 percent of CD4 and 30 percent of CD8 T-cells for more than two cell divisions, whereas cytokine cocktail treated DC induced proliferation of 12 and 11 percent of CD4 and CD8 T-cells, respectively.

**Conclusions:**

The clinically approved compound OK432 has interesting properties that warrants its use in DC immunotherapy and should be considered as a potential immunomodulating agent in cancer immunotherapy.

## Background

Dendritic cells (DC) are a pivotal part of the immune system, bridging the innate and adaptive immune response. After receiving maturation stimuli such as inflammatory cytokines, direct T-cell stimulation or recognition of pathogen-associated molecular patterns (PAMP), the DC up-regulate the surface expression of major histocompatibility complex (MHC) class II as well as a number of co-stimulatory markers [1]. During maturation the DC shift role from antigen up-take to antigen presentation on MHC and migrate to secondary lymphoid organs where the DC stimulate T-cells with the appropriate T-cell receptor. Much work has been carried out to utilise the unique features of DC in clinical applications as *ex vivo *generation of DC has become standard practice [2,3]. Especially, their role in the treatment of malignant neoplasms is one of the areas in which DC show great promise, due to their unique capacity to activate naïve T-cells [4-8].

Immunotherapies with the aid of DC have been shown to be a safe and non-reactogenic way to improve the immune response towards cancer [4-8]. However, it is clear that the immune responses achieved so far have not reached its theoretical potential and DC based therapies have not yet become a standard care of treatment [9]. A recent meta-study found that around 30% of 338 melanoma patients treated with matured DC had either complete response (CR), partial response (PR) or stable disease (SD) [10]. Of the immunological parameters, particularly the induction of antigen specific T-cells was found to be predictive of a positive outcome (CR, PR and SD).

The *ex vivo *protocol published by Jonuleit *et al*. has been hailed as the 'gold standard' in DC maturation [11]. The combination of interleukin (IL)-1β, IL-6, tumour necrosis factor (TNF)-α and prostaglandin E_2 _(PGE_2_) has enabled researchers to produce *ex vivo *matured good manufacturing practice (GMP)-grade DC in large numbers and good viability. This protocol, however, has its deficiencies as the resulting immune response is not optimal for therapeutic cancer vaccination, particularly with its lack of IL-12p70 production [12]. To correct these deficiencies, a number of DC maturation protocols and a range of DC stimuli has been tested for use in cancer immunotherapy [13].

The most critical aspect of DC in conjunction with clinical therapies is the T-cell stimulating capacity of the DC. The DC must be functionally mature in order to induce T-cell activation and not T-cell anergy. Furthermore, the DC must have the ability to migrate to the secondary lymphoid organs to present antigens and induce the effector arm of the immune system. Alternatively, the DC must be able to attract the T-cells to the site of DC injection by secreting signal substances like CCL21 and subsequently induce a T-cell response [14].

In search of better maturation stimuli, we investigated the low-virulence strain of penicillin-killed *Streptococcus pyogenes *(OK432) first described by Okamoto and colleagues [15]. OK432 is available as a licensed drug (trade name, Picibanil) that has already been proven to have little side-effects and has been used efficiently to treat a variety of tumours [16,17]. A meta-study showed significantly better survival with chemotherapy combined with OK432 treatment [18]. The effect of OK432 in cancer patients is not adequately explained, but *in vitro *cultures of OK432 and DC induced the production of inflammatory cytokines [19] and a Th1 like type of response [20,21]. Sato *et al*. [22] did analyse the effect of OK432 on cells from 4 cancer patients with different tumours. Moreover, a small number of patients with glioma enrolled in a phase I/II trial received OK432 treated dendritic cells with promising results [23]. However, additional information is needed to conclude about the effect of OK432 on DC. This is especially important as cancer patients have an inflammatory tumour micro-environment that negatively affects the immune system that also vary between cancer types and progression [Reviewed in [24]].

We used OK432 as a maturation stimulus for DC, both alone in different concentrations or in combination with the Jonuleit cytokine cocktail and compared it to the cytokine cocktail alone in order to understand the effect of OK432 on DC and its possible use in a clinical setting. We found increased secretion of a number of chemokines and cytokines that would result in a local inflammation stronger than that seen after maturation with the cytokine cocktail. Furthermore, the important cytokines IL-15 and IL-12p70 were detected after maturation of DC with OK432, as well as an up-regulated expression of CD40 and a marked increase in T-cell stimulatory capacity. The clinically approved OK432 has interesting properties and should be considered for further DC cancer immunotherapy.

## Methods

### DC generation

DC were generated from monocytes isolated from buffy coat preparations from healthy blood donors (Blood Bank, Haukeland University Hospital, Bergen, Norway) as described [25]. Briefly, peripheral blood mononuclear cells were separated by a density gradient centrifugation and the monocytes were then isolated by plastic adherence. Some experiments were performed with negatively isolated monocytes from buffy coat blood using the Dynabeads^® ^Untouched™ Human Monocytes (Invitrogen, Carlsbad, CA) following the manufacturer's instructions. The monocytes were cultured with IL-4 (20 ng/ml; Immunotools, Friesoythe; Germany) and GM-CSF (100 ng/ml; Immunotools, Friesoythe; Germany) in RP10 medium (RPMI 1640 (Cambrex Bioscience, Verviers, Belgium) with 10% FCS (PAA, Pasching, Austria); 100 units/ml penicillin and 100 μg/ml streptomycin (Sigma-Aldrich, St. Louis, MO)) for 5-6 days to generate immature DC. Cytokines were replenished every 2-3 days. The maturation of dendritic cells was performed for 24 hours with a mix of stimuli (IL-1β, 10 ng/ml; IL-6, 1000 U/ml; TNF-α 10 ng/ml (all from Immunotools, Friesoythe; Germany) and PGE_2_, 1 μg/ml (Sigma-Aldrich, St. Louis, MO) or with OK432 (Picibanil, Chugai Pharmaceutical Co. Ltd, Tokyo, Japan), or a combination of stimuli. The dose of OK432 was given in Klinische Einheit (KE), where 1 KE equals approximately 0.1 mg. Two concentrations of OK432 were tested, 0.1 and 0.01 KE.

### Flowcytometry

Immunostaining was performed as described previously [25]. Briefly, cells were incubated with a titrated amount of antibodies for 10 minutes at room temperature before the cells were washed and immediately analysed on a FACSCanto I cytometer (BD Biosciences, Heidelberg, Germany). All subsequent analyses and gating were done with the FlowJo software (Tree Star, Ashland, OR). One percent false-positive events were accepted in the isotype controls. The antibodies used were CD1-PE (NA1/34-HLK), CD8-PE (LT8), CD14-FITC (UCHM1), HLA-DR-APC (HL-39), CD38-Alexa Fluor 647(AT13/5), CD86-FITC (BU63), CD83-PE (HB15e), CD80-APC (MEM-233), CD40-FITC (LOB7/6), all from AbD Serotec (Düsseldorf, Germany); CCR7-PE, (150503) from R&D Systems (Minneapolis, MN); CD4-APC (MEM-241), CD16-PE (LNK16), CD56-APC (MEM-188) from ImmunoTools, Friesoythe, Germany; and CD3-PerCP-Cy5.5 (UCHT1) from BD Biosciences (San Jose, CA).

### Mixed lymphocyte reaction (MLR)

Allogeneic peripheral blood mononuclear cells (PBMC) depleted for monocytes were thawed and allowed to rest for 24 hours before being labelled with CFDA-SE (Invitrogen, Carlsbad, CA) according to the manufacturer's instructions. Two hundred thousand CFDA-SE labelled lymphocytes were then co-cultured with twenty or forty thousand extensively washed, irradiated, allogeneic matured DC. At the start of the co-culture 50 U/ml of IL-2 and 10 ng/ml of IL-7 (both Immunotools, Friesoythe; Germany) were added to ensure CD4 and CD8 T-cell survival. After 5 days the cells were harvested, stained for CD4 and CD8 for T-cell and CD16 and CD56 for NK cell analysis using a FACS Canto I flowcytometer. The gating strategy included exclusion of cell doublets.

### Chemotaxis assay

After maturation, the DC were harvested and washed extensively. 50 000 live cells were added to the upper chamber of an 8 μm transwell 96-well plate (Corning Lifescience, Lowell, MA) and left to migrate towards CCL19 (100 ng/ml, Immunotools, Friesoythe; Germany) for 16 hours at 37°C, 5% CO_2 _humidified atmosphere. The migrated cells were mixed with a known concentration of beads, counted with a FACSCanto I cytometer, and presented as a percentage of the total number of cells added to the upper well.

### Cytokine determination

To determine the concentration of cytokines and chemokines from the conditioned medium after maturation of the DC, aliquots of the supernatants were stored at -20°C. All samples were tested simultaneously in a 25-plex Luminex assay cytokine and chemokine kit (Invitrogen, Carlsbad, CA) and run on a Luminex 100 System (Luminex Corporation, Austin, TX) according to the manufacturer's instructions. A sandwich ELISA was run separately for IL-12p70 (BioLegend, San Diego, CA), TGF-β1 (R&D Systems, Minneapolis, MN) and IL-23 (eBioscience, San Diego, CA) according to the manufacturer's instructions.

### Statistical analyses

To compare the effect of the stimuli on the DC, group wise analyses were performed with Mann-Whitney non-parametric test. Significance was set at p < 0.05. For the correlation examination, a bivariate analysis shown by the Spearman coefficient (ρ) and the two-tailed test of significance was calculated. All statistical calculation was done with SPSS 15 (SPSS, Chicago, IL) and the correlation heat maps were generated with the iVici program http://michnick.bcm.umontreal.ca/ivici/index.html.

## Results

### OK432 treatment of DC results in maturation and induces higher levels of CD40 compared to the cytokine cocktail

**Figure 1:**
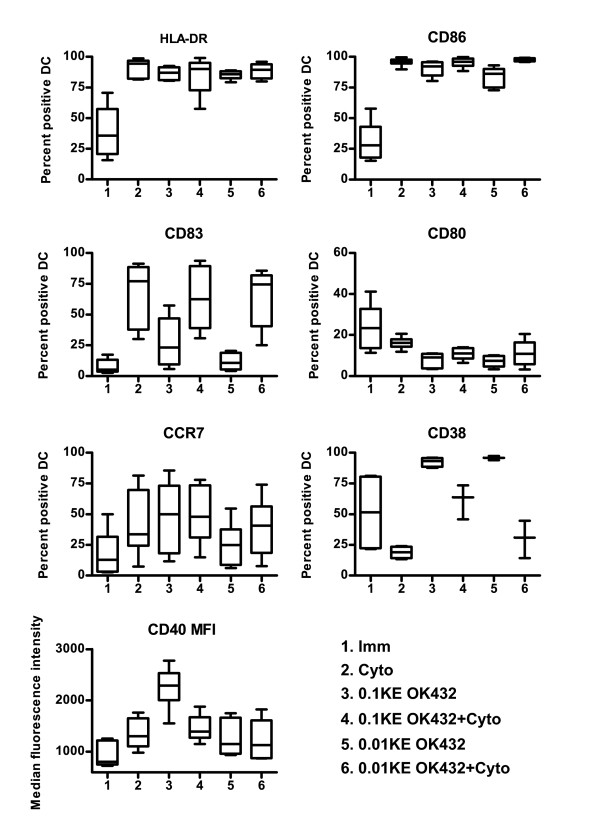
**DC stimulated with OK432 are phenotypically mature with high expression of CD40**. The number of percent positive cells is shown for all surface markers, except CD40, which is displayed with the median fluorescence intensity (MFI) as all groups of DC showed more than 97 percent cells positive for CD40. The number of donors is six for all markers except CD38 where the number of donors is 3 for key 4-6. The distribution is shown by the max/min as well as the 25, 50 and 75 quartiles. Figure key: 1) immature DC (Imm), 2) cytokine cocktail treated DC (Cyto), 3) 0.1KE OK432 treated DC (0.1KE OK432), 4) 0.1 KE OK432 + cytokine cocktail treated DC (0.1KE OK432+Cyto), 5) 0.01 KE OK432 treated DC (0.01KE OK432) and 6) 0.01KE OK432 + cytokine cocktail treated DC (0.01KE OK432+Cyto).

In order to elucidate if the apparent effect seen by the clinical use of OK432 in treatment of tumours is mediated via DC, we first generated monocyte derived immature DC. After a 24-hour maturation stimulus with either 0.1 KE OK432 or cytokine cocktail, both populations had a clear (p < 0.05) up-regulation on the level of HLA-DR and CD86 compared to the immature DC (Figure 1). A reduced dose of 0.01 KE OK432 also induced a statistically significant, but lower, increase of HLA-DR and CD86 compared to immature DC.

Maturation of DC with the cytokine cocktail led to higher levels of the co-stimulatory markers CD80 and CD86 (p < 0.02) and activation marker CD83 (p < 0.05) on the cell surface than OK432 treated DC. The use of cytokine cocktail and OK432 led to a phenotype resembling that of cytokine cocktail alone. The level of CD40 together with the other co-stimulatory molecules is important for an adequate stimulation of T-cells [26]. OK432 induced a 75 percent higher median fluorescence intensity (MFI) of CD40 on the surface of the DC compared to cytokine cocktail alone (p < 0.01) or cytokine cocktail and OK432. The MFI values for all markers are given in additional file 1. The level of CCR7, a marker important for migration, was also investigated. However, the MFI was only modestly increased after OK432 treatment and the difference was not statistically significant.

CD38 is a cyclic ADP ribose hydroxylase involved in the regulation of intracellular Ca^2+ ^[27]. Its surface expression on DC has been reported to have a range of different functions in migration, survival, IL-12p70 production [28] and support Th1 skewing of the immune response [29]. OK432 up-regulated CD38 on mature DC (p < 0.05), in contrast to the cytokine cocktail, which reduced CD38 expression compared to immature DC (not statistically significant).

### Cytokine cocktail induces the highest level of chemotaxis

**Figure 2:**
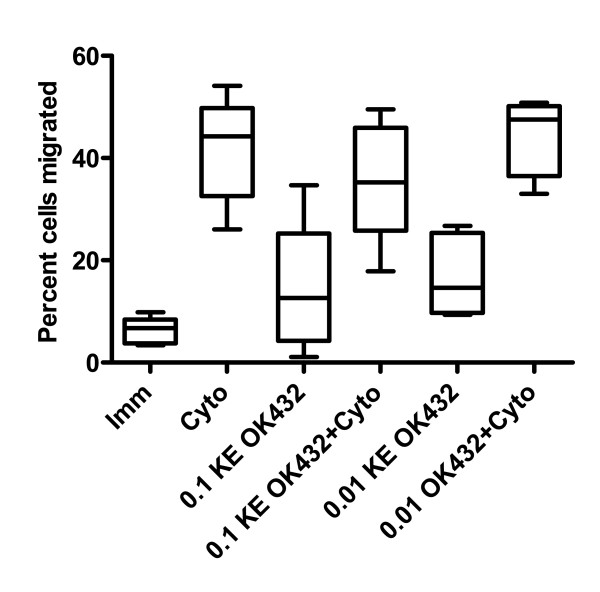
**DC maturated with OK432 show less migration towards CCL19 compared to cytokine cocktail treated DC**. After 24 hours stimulation with the indicated stimuli, the migratory capacity of the DC populations towards CCL19 was analysed in a transwell migration assay for 16 h. The chemotaxis is presented as the percentage of cells that migrated to the bottom well relative to the input number of DC in the upper well. The number of donors is six. The distribution is shown by the max/min as well as the 25, 50 and 75 quartiles. See figure 1 for figure key.

One possible reason for the promising clinical effects of OK432 might be an improved ability of DC to migrate to sites of inflammation. The differentially matured DC were therefore tested in a transwell migration assay to investigate if the different maturation stimuli resulted in changes in DC mobility towards CCR7 ligand CCL19 (Figure 2). This chemokine has been shown to be at least one order of magnitude more potent than CCL21 for directing DC chemotaxis in a murine model [30]. DC treated with the cytokine cocktail migrated best, followed by a combination of cocktail and 0.01KE OK432, which was slightly better in migration than the cytokine cocktail and 0.1KE OK432. Of the DC that received only OK432, the group that received 0.01KE OK432 migrated to about the same extent as the DC matured with 0.1KE OK432. Unlike previous reports, the level of CD38 as percent positive cells or as MFI (Table 1) was not linked to better performance in the chemotaxis assay [31].

**Table 1:** Parameters significantly associated with DC chemotaxis ex vivo

	Spearman coefficient, ρ	P-value
IL-1β	-0.442	0.045

IL-8	0.472	0.023

CXCL9	-0.483	0.020

HLA-DR^a^	0.365	0.044

HLA-DR ^b^	0.400	0.026

CD86^a^	0.788	0.000001

CD86 ^b^	0.641	0.0001

CD83^a^	0.763	0.000001

CD83 ^b^	0.412	0.021

^a ^measured as percent positive cells; ^b ^measured as median fluorescence intensity

### OK432 elicits a pro-inflammatory cytokine and chemokine profile

**Figure 3:**
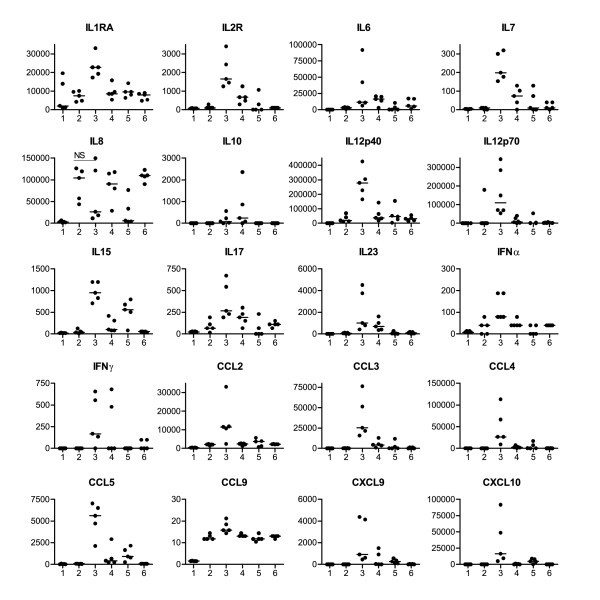
**OK432 stimulated DC secrete higher quantities of cytokines and chemokines compared to cytokine cocktail treated DC**. The DC were stimulated for 24 hours and the amount of cytokines/chemokines secreted were analysed using a 25-plex bead assay and a sandwich ELISA for IL-12p70, IL-23 and TGF-β1. Only cytokines and chemokines with statistically (group wise analyses with Mann-Whitney) different concentrations between DC stimulated with cytokine cocktail and 0.1 KE OK432 are shown, with the exception of IL-8. No significant difference was found for IL-1β, IL-2, IL-5, IL-13, TGF-β1 and TNF-α. GM-CSF and IL-4 were part of the added cytokines and therefore not considered in the analysis. The concentrations are given in pg/ml. See figure 1 for figure key.

Production and release into the medium of 28 cytokines and chemokines was analysed by a multiplex luminex assay and sandwich ELISA (Figure 3). OK432 treatment induced the secretion of a range of pro-inflammatory cytokines that were not detected after maturation with the cytokine cocktail. IL-1RA, -2R, -6, -7, 10, -12p40, -12p70, -15, -17, -23, IFN-α, and IFN-γ and all the chemokines tested (CCL3/MIP1α, CCL4/MIP1β, CXCL10/IP10, CXCL9/MIG, CCL11/Eotaxin, CCL5/RANTES and CCL2/MCP1) were significantly (p < 0.05) up-regulated after 24 hour stimulation with 0.1 KE OK432 compared to the cytokine cocktail. TNF-α was borderline significantly higher (p = 0.056) in supernatants from cells matured with OK432, even though TNF-α was a part of the cytokine maturation cocktail and not added to the cultures receiving OK432. The reduced concentration of 0.01KE OK432 resulted in from a 2-fold increase to almost no detection of the secreted cytokines and chemokines compared to the cytokine cocktail, therefore a reduced concentration of OK432 did not achieve satisfying maturation.

In general, using combinations of the cytokine cocktail and OK432 resulted in a similar, but lower, level of cytokines and chemokines as compared to the cytokine cocktail alone. With regard to IL-12p70 and IL-15, the production was lost or greatly reduced (> 95 percent) when both stimuli were combined and thus the combination of cytokine cocktail and OK432 was of lesser interest. To exclude the possibility of inducing a regulatory T-cell response, the amount of TGF-β1 was measured and found not to be increased after OK432 treatment (data not shown).

### Surface expression of maturation markers predict elevated chemotaxis

**Figure 4:**
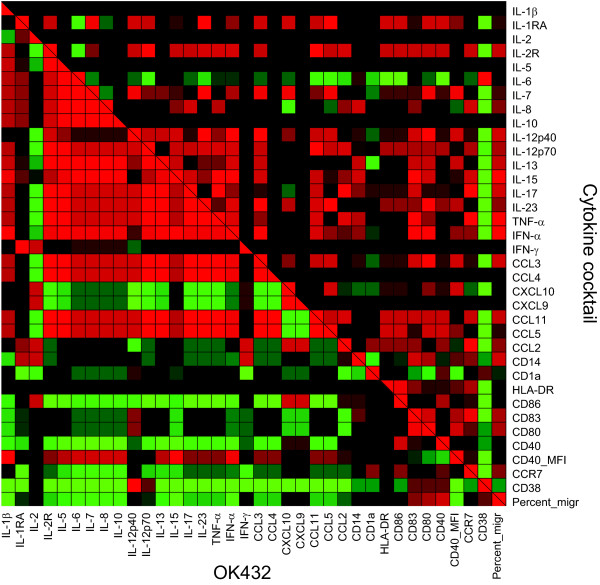
**The effect of OK432 is clearly different from the effect of the cytokine cocktail**. To reveal the relationships between the parameters tested and the similarities between DC matured with the cytokine cocktail and OK432 for 24 hours, a bivariate correlation analysis was performed and the Spearman coefficient was plotted in a heat map. The Spearman coefficient is in the interval from -1 (negative correlation, shown in green) to 0 (no correlation or no data, shown in black) to +1 (positive correlation, shown in red). Top right shows the correlation for the cytokine cocktail treated DC and lower left side shows correlation for OK432 treated DC. For the surface markers the percentage of positive cells is displayed, except CD40, which is shown with the MFI. The correlation does not change when including the MFI for the rest of the flow cytometry markers. The migration is displayed with the percentage of migrated DC. Five independent experiments are included.

In order to understand the differences in maturation induced by the stimuli tested, a correlation analysis was undertaken to elucidate which of the parameters were regulated in a similar manner. The goal was two-fold; firstly to investigate which parameter predicted good migratory capacity for all matured DC and secondly; to envisage the differences between OK432 and the cytokine cocktail in general. The parameters included in the analysis were the percentage of positive cells for the surface markers tested and the MFI of CD40, the concentration of 27 chemokines and cytokines in conditioned medium from the matured DC and the percentage of DC that migrated towards CCL19. The analysis revealed that IL-8 production and the surface expression of HLA-DR, CD86 and CD83 were positively correlated with increased chemotaxis ability, shown by the Spearman coefficient (ρ) (Table 1). CXCL9 and IL-1β were significantly negatively correlated with chemotaxis and the same trend was seen for CD38 but without statistical significance (ρ = -0.418, p = 0.075). CCR7 expressed as percent positive cells was only weakly associated with chemotaxis (ρ = 0.185, p = 0.320), this was also true when testing CCR7 MFI (ρ = 0.240, p = 0.194). To relate the different stimuli to all the parameters tested, a correlation analysis was performed and the correlation matrixes for the cytokine cocktail and OK432 are shown in a heat map (Figure 4) to allow an appreciation of the differences the two maturation stimuli have on the parameters tested. The figure clearly demonstrates that the cytokine cocktail and OK432 have distinctly effects on both the surface markers and secreted cytokines and chemokines.

### Dendritic cells matured with OK432 are best in allogeneic T-cell stimulation

**Figure 5:**
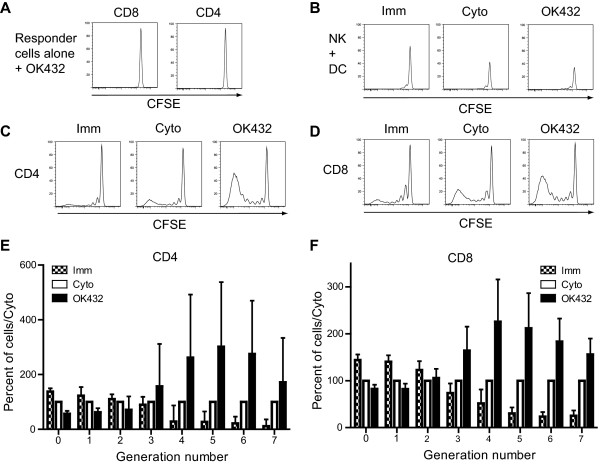
**DC matured with OK432 are exceeding the cytokine cocktail treated DC in allogeneic T-cell stimulation**. DC were matured for 24 hours with the indicated stimuli, washed, irradiated, and 2×10^4 ^DC were co-cultured with 2×10^5^CFSE labelled allogeneic leukocytes that had the monocyte population removed, for 5 days. The cells were harvested, stained for CD4 and CD8, and analysed on a flowcytometer. Panel A show the negligible effect the 0.1 KE OK432 compound has on the T-cell proliferation in an allogeneic leukocytes culture in the absence of direct DC stimulation. Panel B shows the effect immature, cytokine cocktail and OK432 stimulated DC have on the total NK cell population (CD3^-^CD16^+^CD56^+/-^) within an allogeneic leukocytes culture that had the monocyte population removed. Similar results were found using forty thousand DC in the co-culture (data not shown). Histograms from one experiment are shown in C and D. Panel E and F show the median percentage compared to cyto (from the same donor) set at 100 percent plus interquartile range in division number from 0 to 7, for CD4 and CD8 positive cells. (n = 4). DC treated with OK432 induced more proliferation of both CD4 and CD8 positive cells and had a high proportion of cells with the maximum detectable divisions. Figure key: Imm: Immature DC; Cyto: cytokine cocktail treated DC; OK432: 0.1KE OK432 treated DC.

As OK432 induced high surface expression of CD40 as well as high levels of cytokines and chemokines, we next tested if this increased inflammatory milieu surrounding matured DC would be of functional consequence in T-cell induction. Combinations of OK432 and cytokine cocktail or reduced concentrations of OK432 were not tested in MLR as they did not elicit adequate maturation levels, chemotaxis and reduced level of secreted inflammatory cytokines and chemokines by the DC. First, any direct stimulation effect of the T-cells by residual OK432 was ruled out as culturing T cells alone with 0.1 KE OK432 resulted in no T-cell proliferation (Figure 5A). The total NK cell compartment (CD3^-^CD16^+^CD56^+/-^CFSE^+^) showed no remarkable differences between the DC stimuli (Figure 5B).

Matured DC were co-cultured with allogeneic T-cells in a PBMC culture depleted for monocytes and the level of induced proliferation was measured by CFSE-dilution (Figure 5C, D). Immature DC induced a low level of proliferation, with around 10 percent of CD4 and 6 percent CD8 positive lymphocytes having undergone more than 2 divisions after five days of co-culture. In comparison, using cytokine cocktail treated DC around 23 percent of CD4 and 28 percent of CD8 positive T-cells had undergone more than 2 divisions. OK432 treated DC induced extensive T-cell proliferation with more than 50 percent of CD4 and more than 40 percent of CD8 positive cells having undergone more than two cell divisions; most had undergone five or more cell divisions. Importantly, OK432 treated DC stimulated 2-3 fold more CD4 positive T-cells in later generations (generation 4-7) and about 2-fold more CD8 positive T-cells in later generations than cytokine cocktail treated DC (Figure 5E, F).

## Discussion

The use of OK432 in different clinical applications has resulted in positive clinical outcomes over a period of several decades [15-17,32]. The immunomodulating mechanism behind OK432 has not yet been conclusively determined, but a general boost of the immune system seems evident. OK432 and other microbial products are sensed by PAMPs which results in an immune activation. This primes the immune system, as demonstrated by the effect of intralesional OK432 injections into lymphangioma [17]. These clinical experiences led us to investigate if OK432 might also be of use in other forms of immunomodulation. Although other researchers have worked with OK432 and DC, a combination of OK432 and the cytokine cocktail has not been thoroughly investigated after DC stimulation. The effects of OK432 dosage and the combination with cytokine cocktail on DC maturation and the full range of chemokines and cytokines that we present have not been reported before.

Elicitation of an anti-tumour immune response is the aim of cancer immunotherapy and for all DC based vaccine approaches the immune response relies heavily on the immune-stimulatory capacity of the DC population being used. This quality is above all dependent of the choice of maturation stimuli, which in the clinical protocols is often a cocktail of cytokines, first described by Jonuleit *et al*. [11]. One challenging aspect with this protocol is the addition of PGE_2_, which is thought to be necessary to ensure DC survival and migration, and also induce expression of OX40L [33]. However, the production of IL-12p70, which is crucial for initiation of the required Th1 type of response and the induction of high quality CTL, is lost by the use of the cytokine cocktail as also we have demonstrated, and this is due to the addition of PGE_2 _[34,35]. PGE_2 _treated DC have also been found to attract T-regulatory cells, which would be negative for induction of an immune response [36]. Thus, Palucka *et al*. summarised their work by emphasising that the next generation of DC immunotherapy should elicit high concentrations of IL-12p70 and IL-15, in addition to surface expression of co-stimulatory molecules on the DC [37].

Stimulation with OK432 increased the secretion of nearly all cytokines and chemokines tested, suggestive of an increased local inflammation when used in immunotherapy. This included also IL-15 and IL-12p70 which have been found to be important for the proliferation of NK cells and IFN-γ production by DC, and both aspects are of importance in generating a potent Th1 type of immune response [38]. Endogenous IL-15 production at the same time as antigen priming has been shown to be central to the priming and longevity of the CTL response [39,40]. From our data it is also clear that no synergistic effects could be detected as combinations of the cytokine cocktail and OK432 resulted in a greatly reduced IL-12p70 and IL-15 production. We cannot, however, rule out that a sequential stimulation might have changed this cytokine profile, as demonstrated with the TLR3 agonist poly I:C and the cytokine cocktail [41] and this might be analysed in future studies. In a murine study, IL-7 was found to boost vaccine-induced antitumour immunity by increasing both T-cell effector function and the NK cell numbers [42], but only small differences were detected in NK cell numbers after co-culture of lymphocytes and DC in our experiments. From the presented cytokine and chemokine data it seems evident that the inflammatory milieu surrounding DC treated with OK432 was elevated and in the direction of a Th1 response. Also in wash-out cultures of OK432 treated DC, the IL-12p70 production was sustained at a low level (~300 pg/ml) for at least 48 hours without any stimuli (data not shown). Although that is considerably lower than after 24 hours with OK432 stimulation, it is still remarkable that the DC are able to secrete IL-12p70 after the stimulus is lifted. As OK432 has a good safety profile, it could possibly also be used systemically after DC infusion in patients to prolong and sustain the proinflammatory response. A distinct set of immune responses was detected between OK432 and the cytokine cocktail from the correlation on the measured parameters. This illustrated clearly that OK432 and the cytokine cocktail affected the different parameters measured very differently with OK432 up-regulating more cytokines, chemokines and co-receptors.

When the different matured DC populations were tested in a mixed lymphocyte reaction, the inflammatory response induced by OK432 on DC was sustained in the absence of further stimuli. The secreted cytokines, chemokines and co-stimulatory molecules induced a high proliferation of both CD4 and CD8 T-cells. This is an indication that OK432 induced the DC to become more supportive of T-cell proliferation than the cytokine cocktail demonstrating the potential to stimulate the cell mediated immune response. Importantly, the OK432 stimulated DC were particularly able to stimulate more than five divisions of T-cells, indicative of a robust immune response. Stimulation of DC with OK432 resulted in a marked up-regulation of the important co-stimulatory marker CD40 on the surface of the DC, compared to cytokine cocktail alone, which has not been reported before with the 7 days DC generation protocol. Engagement of CD40 on APC acts to license T-cells and thereby recruiting additional DC and making them more potent in eliciting an immune response [4]. The CD40 pathway indirectly amplifies the T-cell response and thus is important in inducing a good cell mediated immune response [26], which may also be partly the reason why OK432 stimulated DC induced such a high level of T-cell activation.

The ability to migrate towards CCL19 differed significantly between the different stimuli. DC matured with OK432 and cytokine cocktail had similar levels of CCR7 expression; OK432 treated DC did nevertheless migrate less efficiently than DC treated with the cytokine cocktail. Sakakibara *et al*. [20] found OK432 DC to be better at chemotaxis than the cytokine cocktail DC, but they used less PGE_2 _in the cytokine cocktail than Jonuleit *et al*. [11], which may account for the poorer migration of the cytokine cocktail treated DC. PGE_2 _has been demonstrated to be important for activating the migration machinery, independent of the level of CCR7 [34]. This also illustrates that the *in vitro *chemotaxis of DC towards CCL19 is not determined solely by CCR7, but also by additional mechanisms. Furthermore, it has been suggested that a lower migration of DC could still be sufficient if the DC that migrate *in vivo *were able to induce a good T-cell response with a higher IL-12p70 production than the cytokine cocktail DC [43].

The ADP ribose hydroxylase CD38 has been reported to be a novel marker for mature DC and involved in the migratory capacity of DC [28,29,31] and we demonstrated that OK432 up-regulated this marker. The correlation analysis of all combined mature DC showed, however, that the chemotaxis was clearly related to a number of other maturation markers, particularly CD86 and CD83, whereas no significant correlation was found to markers like CD38, or indeed, to CCR7. Furthermore, CD38 expression seemed to be dependent on the maturation stimuli, rather than the maturation status, as CD38 was down-regulated on DC matured with the cytokine cocktail. Other studies have shown that increased migratory capacity was linked to increased expression of CD38 on the surface of DC, but under maturation conditions without PGE_2 _[28,31]. Conversely, we found that OK432 treatment of DC up-regulated both CD38 and CD83, but was still unable to induce chemotaxis to the same extent as the cytokine cocktail. Initiation of migration is a complex chain of events [44], but at least the level of CD38 induced by the OK432 did not seem to be the critical determinant for the induction of CCL19 directed chemotaxis.

## Conclusions

As for the use of OK432 in cancer immunotherapy, we have demonstrated that OK432 is better than the cytokine cocktail in several critical aspects, i.e. IL-12p70 and IL-15 production, CD40 expression and T-cell stimulation. It would therefore be interesting to verify if this could also be beneficial when these DC are utilised in cancer immunotherapy. In conclusion, OK432 with its demonstrated clinical efficacy and safety profile is an attractive compound also in the context of DC based immunotherapy.

## Competing interests

The authors declare that they have no competing interests.

## Authors' contributions

AOH planned and executed the experiments and drafted the manuscript. MK participated with the experiments, carried out the cell culture and assisted in analysing the data. RJ participated in the coordination of the study, its conception and design. H-JA initiated the work with OK432 and critically revised the manuscript. SA conceived the study, and participated in its design and coordination and helped to draft the manuscript. All authors read, gave critical input to and approved the final manuscript.

## Supplementary Material

Additional file 1 **Table with MFI data**. Median fluorescence intensities (MFI) of markers shown in Figure 1. Click here for file
